# A genetic screen for *Drosophila* social isolation mutants and analysis of *sex pistol*

**DOI:** 10.1038/s41598-021-96871-x

**Published:** 2021-08-30

**Authors:** Mark Eddison

**Affiliations:** Howard Hughes Medical Institute, Janelia Research Campus, 19700 Helix Drive, Ashburn, VA 20147 USA

**Keywords:** Social behaviour, Aggression, Behavioural genetics

## Abstract

Prolonged periods of forced social isolation is detrimental to well-being, yet we know little about which genes regulate susceptibility to its effects. In the fruit fly, *Drosophila melanogaster,* social isolation induces stark changes in behavior including increased aggression, locomotor activity, and resistance to ethanol sedation. To identify genes regulating sensitivity to isolation, I screened a collection of sixteen hundred P-element insertion lines for mutants with abnormal levels of all three isolation-induced behaviors. The screen identified three mutants whose affected genes are likely central to regulating the effects of isolation in flies. One mutant, *sex pistol* (*sxp*), became extremely aggressive and resistant to ethanol sedation when socially isolated. *sxp* also had a high level of male–male courtship. The mutation in *sxp* reduced the expression of two minor isoforms of the actin regulator *hts* (adducin)*,* as well as mildly reducing expression of *CalpA*, a calcium-dependent protease. As a consequence*, sxp* also had increased expression of the insulin-like peptide, *dILP5*. Analysis of the social behavior of *sxp* suggests that these minor *hts* isoforms function to limit isolation-induced aggression, while chronically high levels of *dILP5* increase male–male courtship.

## Introduction

Being forcefully deprived of social contact can profoundly alter behavior, negatively impacting somatic and psychiatric health. In fact, objective social isolation and subjective, or perceived, social isolation (i.e. ‘feeling lonely’) is as a significant risk factor for morbidity and mortality as alcoholism, smoking, obesity and high blood pressure^1,2^. Further, the lack of meaningful social relationships is considered a major contributor in the etiology of several psychiatric disorders^3^. Symptoms of social isolation can include heightened anxiety, anger and depression, cognitive rigidity, impaired sleep, increased drug use and a compromised immune response^4^. Individuals differ in their response to social isolation and it is estimated that heritability accounts for approximately 50% of these differences^5,6^. Yet despite the fact that social isolation is increasing in society^7^, especially during these times^8^, we are only beginning to identify which genes regulate susceptibility to its effects^6,9^.

Like humans, most animals are also negatively impacted by forced social isolation. In rodents, social isolation increases measures of anxiety, aggression and cognitive deficits, that vary according to the age at the time of isolation and length of being socially deprived^10,11^. Social isolation in rodents also increases alcohol consumption^12^ and other drug-associated behaviors^13^. Such changes in behavior are likely due to differences in neurogenesis^14^, epigenetics^15^, gene expression^16^, phosphorylation^17^, neuronal morphology^10^, physiology^18^ and neuroendocrine secretion^19^ in isolated individuals. Importantly, social isolation in rodents has been used to model anxiety and depressive disorders^20^, schizophrenia^21^ and drug abuse^13^; diseases that in common have dysregulated emotion and associated behaviors. Isolation also has a profound effect on the behavior of the fruit fly, *Drosophila melanogaster*. Isolated males have increased aggression^22,23^, social interactions^24^, daytime locomotion^25,26^ and resistance to ethanol sedation^27^, while courtship duration and grooming are decreased^24,28^. Like mammals, differences have been documented in the neuronal morphology^29–31^, gene expression^23^, epigenetics^32^ and physiology^33^ of isolated flies, raising the possibility of conserved mechanisms that act to alter behavior in response to being isolated.

Here I describe a forward genetic screen aimed to identify candidate genes central to the regulation of isolation-induced behaviors, such as the recently described role of the Tac2 neuropeptide in mice^34^. The screen identified three mutations and candidate genes that affected isolation-induced changes in aggression, locomotor activity and ethanol resistance. Importantly, all three behaviors were normal when the mutants were socially housed, suggesting the candidate genes specifically mediate effects of isolation. Here, I describe an in-depth analysis of one mutant, *sex-pistol* (*sxp*), demonstrating that its mutation affects at least two genes that separably regulate the level of male–male aggression and male–male courtship.

## Results

### A forward genetic screen for social isolation mutants

**Figure 1:**
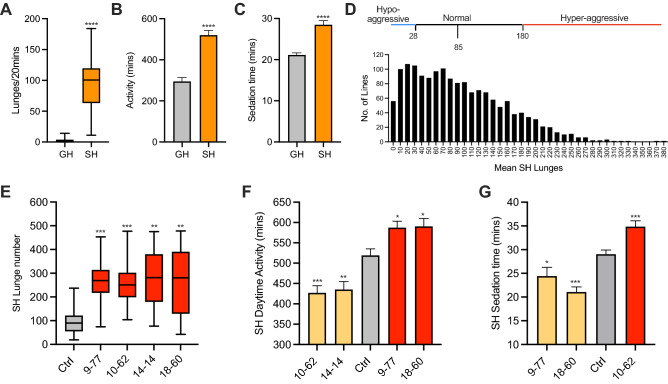
A forward genetic screen for social isolation mutants. (**A**–**C**) Social isolation increases aggression, daytime locomotor activity and ethanol sedation resistance in male flies (****p < 0.0001, Mann–Whitney U test). (**A**) Aggression was measured as the number of lunges in 20 min (n = 24); (**B**) Total daytime activity (min) was measured in the DAM assay (n = 29); (**C**) Ethanol resistance was measured as median sedation time (min) (n = 18). (**D**) A histogram of the aggression screen of 1606 P-element lines showing frequency of lines with mean lunges. Mean and median lunge number was approx. 85 lunges (SD ± 65). Mutants with a lunge mean < 28 (1 SD below mean, blue) were considered hypo-aggressive and lunge mean > 180 (1.5 SD above mean, red) hyperaggressive. (**E**) SH lunge number of activity and aggression mutants (***p < 0.0005, **p < 0.0066, Kruskal–Wallis Test with Dunn’s MCT, n = 15–24). (**F**) SH activity of activity and aggression mutants (hyperaggressive and hypoactive (yellow), hyperaggressive and hyperactive (red) (*p < 0.0279, **p < 0.0048, ***p < 0.0003, One-Way ANOVA with Dunnett’s MCT, n = 30–46). (**G**) SH ethanol sedation times of 3 activity and aggression mutants (*p < 0.0481, ***p < 0.0009, One-Way ANOVA with Dunnett’s MCT, n = 6–26).

Male flies socially isolated for five days show obvious, robust, and quantifiable increases in aggression, daytime locomotion, and resistance to ethanol sedation (Fig. 1A–C)^23,25–27^. These changes in behavior suggest that isolation induces an internal state change that modifies multiple behaviors^35^. To identify genes that may regulate this state, I sought to identify mutants with abnormal levels of isolation-induced aggression, activity and ethanol resistance. The strategy (Supplementary Fig. S1) was to first screen for aggression-related mutants, then screen aggression mutants for abnormal locomotor activity. Aggression and activity mutants were then tested for ethanol resistance. Any gene affected in a mutant which had abnormal levels of all three behaviors when single-housed (SH), but not group-housed (GH), was therefore likely central to regulating isolation-induced behavior.

To identify aggression mutants, I screened the Heberlein collection of 1606 lines that each have a single random insertion of a P*-GAL4* element that affects the level of gene transcription. Male flies were isolated for five days, and aggression measured (Fig. 1D). Aggression was quantified as the total number of lunges performed between two males of the same genotype in 20 min (see Methods). I defined a normal level of aggression based on the mean and median of the whole population (~ 85 lunges/20 min). To identify aggression mutants at the ends of the distribution, lines were considered hyperaggressive if their lunge mean was greater than 1.5 SD above the mean (180 lunges/20 min) and due to the left skew in the distribution, hypo-aggressive if their lunge mean was less than 1 SD below the mean (28 lunges/20 min). 

In total, 15% of the population (244 lines) met these criteria and were backcrossed to the genetic background (*wBerlin*) for five generations to remove unlinked modifiers. After retesting, 183 lines (75%) lost their aggressive phenotype and 61 lines remained mutant (15 hypo-aggressive and 46 hyperaggressive), demonstrating a significant influence of unlinked modifiers on SH aggression. Using inverse PCR, the P-element insertion site in all aggressive mutants was mapped (Supplementary Table S1). Importantly, insertion sites included several gene loci previously associated with aggression (e.g. *lama, RtnL1* and *kis*^36–38^), validating the screen. To be sure being isolated was the primary cause of increased aggression, hyperaggressive lines were also tested for aggression when GH. Here, 4/46 hyperaggressive lines were also aggressive when GH and not considered further (Supplementary Table S1).

Next, using the *Drosophila* Activity Monitor (DAM), I measured the daytime locomotor activity of SH aggression mutants when GH and SH (Supplementary Table S2). The majority (37/61) had normal activity in both housing conditions and not considered further. Another fourteen lines had abnormal activity when GH and were also ruled out. In summary, after testing SH mutants in both aggression and activity assays four SH hyperaggressive mutants also had abnormal activity when SH, but not GH (Fig. 1E, F, Supplementary Table S3). Two hyperaggressive mutants were also hyperactive when SH, suggesting these lines (*9-77* and *18-60*) were more sensitive to isolation. Curiously, the other two hyperaggressive mutants were hypoactive when SH (*10-62* and *14-14*), suggesting a more complex relationship between candidate gene and isolation-induced aggression and activity.

Next, I tested 3 SH aggression and activity mutants for abnormal resistance to ethanol sedation (Supplementary Table S4). While all had normal GH ethanol resistance, only SH *10-62* was more ethanol resistant than SH control (Fig. 1G). While there was no correlation in the direction of aggression and ethanol resistance, there was an inverse correlation between activity level and ethanol resistance.

In summary, this screen identified three mutants that had abnormal levels of aggression, locomotor activity and ethanol resistance when isolated. Therefore, the genes affected in these mutants are likely central in regulating the effects of isolation. However, no mutant affected all three isolation-induced behaviors in the same direction, suggesting the relationship between each gene and these behaviors is complex. I choose to examine line *10-62* in more detail*,* as its phenotype was the most robust and its P-element is inserted in *hts* (aka adducin), an actin regulator that is known to regulate synaptic stability across species^39,40^. Of note, synapse stability is also affected by social isolation^31^, actin dynamics regulates isolation-induced social dominance behavior in mice^41^, and is strongly implicated in mental health disorders in humans^42^.

### *10*-*62* has an excessive level of social behaviors

**Figure 2:**
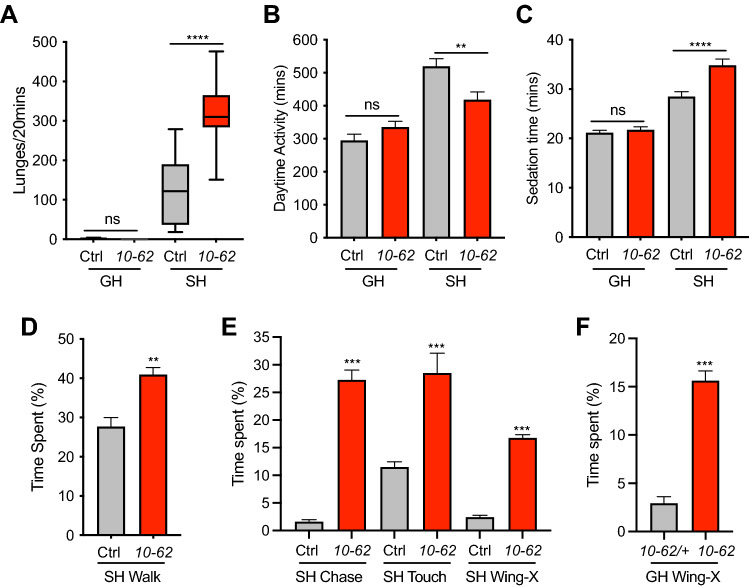
*10-62* has an excessive level of social behaviors. (**A**) GH *10-62* males were as unaggressive as GH control (p = 0.991), but hyperaggressive when SH (****p < 0.0001; 2-Way ANOVA with Sidak’s MCT, n = 12–24). (**B**) *10-62* males had normal locomotor activity when GH (p = 0.2892), but decreased activity when SH (**p < 0.0019; 2-Way ANOVA with Sidak’s MCT, n = 29–32). (**C**) GH *10-62* males had normal resistance to ethanol sedation (p = 0.8609), but increased ethanol resistance when SH (****p < 0.0001; 2-Way ANOVA with Sidak’s MCT, n = 13–18). In the fly bowl, SH *10-62* males showed increased time spent (as %) (**D**) walking (**p < 0.0019, n = 8, Mann–Whitney Test) and (**E**) chasing, touching, and performing single wing-extensions (Wing-X) (***p < 0.0002); n = 8, Mann–Whitney Test). (**F**) GH *10-62* males also showed increased male–male courtship behavior in the fly bowl (***p < 0.0002); n = 8, Mann–Whitney Test).

While GH *10-62* males had normal levels of aggression, locomotor activity and ethanol resistance, SH *10-62* males were hyperaggressive, less active, and more resistant to ethanol sedation compared to the SH control (Fig. 2A–C). *10-62* fecundity appeared normal, and males had no obvious morphological defects in brain structure (Supplementary Fig. S2). One notable difference was that *10-62* body mass was ~ 5% heavier in both GH and SH conditions (Supplementary Fig. S2). Given its elevated SH aggression and SH ethanol resistance, it seemed counterintuitive that SH *10-62* males had decreased daytime locomotor activity (Fig. 2B). A potential reason for this unexpected finding is that the DAM assay is a single fly assay, while both the aggression and ethanol resistance assays are not. Therefore, I measured the locomotor activity of SH *10-62* in the fly bowl*,* a relatively large arena^43^ where groups of twenty SH males interact. Interestingly, using the Janelia Automatic Animal Behaviour Annotator^44^ to quantify behavior I measured a significant increase in the time SH *10-62* spent walking (Fig. 2D)*.* More strikingly, I also observed an increased time spent chasing and touching, as well as performing single-wing extensions, that are usually reserved for male–female courtship (Fig. 2E, Supplementary Movies S1, S2). Of note, the increased male–male courtship of SH *10-62* was also observed in GH *10-62* males (Fig. 2F, Supplementary Fig. S2), suggesting the excessive male–male courtship of *10-62* was independent of isolation. Indeed, SH did not significantly increase male–male courtship in control flies (Supplementary Fig. S2). Therefore, in the aggression chamber, which is relatively small and contains food, SH *10-62* males were more aggressive than normal, while in the fly bowl chamber, which is relatively large and has an absence of food, they were more sexual than normal. Sex or genotype preference was not tested, though in the fly bowl, *10-62* males also spent more time wing-flicking (Supplementary Fig. S2), a defensive behavior possibly initiated in response to other males’ courtship advances.

In summary, when alone in the DAM assay SH *10-62* males had decreased activity, but when grouped with other SH *10-62* males in the fly bowl they had increased activity. This increased activity was likely driven by its excessive male–male courtship, which was independent of housing regime. Since the behavioral repertoire of *10-62* males included excessive levels of sex, aggression, and ethanol resistance, I named the mutant *sex pistol* (*sxp*) in reference to the anarchistic 70’s punk band.

### *Sex pistol *has reduced expression of *hts* and* CalpA*

**Figure 3:**
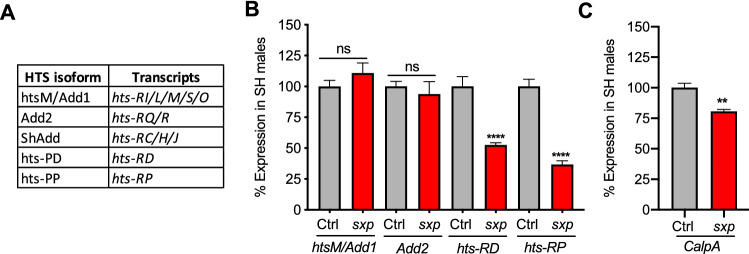
*sxp* Flies have Reduced Expression of Specific *hts* Isoforms and *CalpA*. (**A**) The HTS protein isoforms expressed in males and encoding transcripts. (**B**) By q-PCR, no difference in expression level of *htsM/Add1* or *Add2* transcripts in SH *sxp* (p > 0.05, n = 6) was detected, but there was a significant reduction in *hts-RD* and *hts-RP* (****p < 0.0001; n = 6, Unpaired t-test). (**C**) SH *sxp* also had a mild reduction in *CalpA* expression (**p < 0.0086; n = 3, Unpaired t-test).

The P-element in *sxp* is inserted between the 1st and 2nd exons of *hts* (or *adducin*)*,* a gene which encodes a conserved protein that forms heterodimers or tetramers and is known to regulate actin dynamics and synaptic stability across species^39,40,45^. *Drosophila hts* encodes multiple protein isoforms. Males have five, called Add1/HtsM, Add2, ShAdd, hts-PD^46^ and hts-PP (flybase.org) which mainly differ in the C-terminal domain (Fig. 3A). To decipher if there was any change in level of *hts* transcripts in *sxp*, I performed q-PCR with various *hts* primers on RNA extracted from isolated male heads. Comparing SH control and SH *sxp* males, I did not observe a difference in expression of *Add1/HtsM,* the predominant isoform expressed in the brain^39^, or *Add2* (Fig. 3B). However, *sxp* did have an approximately 50% reduction in *hts-RD* and a 38% reduction in *hts-RP* transcripts. Of note, expression of *hts-RD/RP* in control and *sxp* flies was lower than levels of *Add1* (with an average Ct value of ~ 29 vs 25), suggesting a reduced or more localized expression. Due to sequence similarity, I could not assess if *ShAdd* was affected. I did not detect a difference in any *hts* transcript tested between GH and SH flies, suggesting isolation does not alter *hts* expression in *sxp* or control flies (Supplementary Fig. S3).

As the P-element also sits close (~ 700 bp) to *CalpA*, a calcium-dependent endopeptidase that regulates dendritic pruning^47^, I next checked levels of *CalpA* (with primers common to all four isoforms). Interestingly, SH *sxp* males had a small (approximately 20%) but significant, reduction of *CalpA* expression (Fig. 3C). As with *hts*, *CalpA* expression was unaffected by isolation (Supplementary Fig. S3). In summary, the P-element insertion in *sxp* significantly reduced expression of *hts-RD, hts-RP* and *CalpA*, but neither *hts* nor *CalpA* transcription was altered by isolation. As this analysis was performed on whole male heads, I cannot draw conclusions about gene expression level in sub-populations of cells in the brain or body.

### *hts-RD/RP* limits isolation-induced aggression

**Figure 4:**
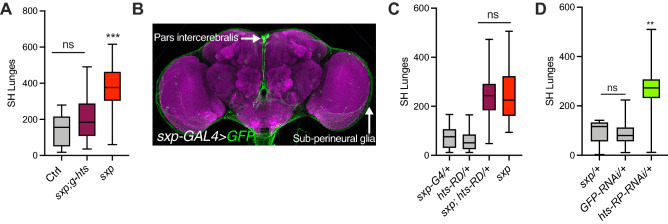
*hts* limits isolation-induced aggression. (**A**) Inclusion of genomic *hts* construct (fTRG-585) restored normal levels of aggression to *sxp* (p > 0.05, n = 21–24, Kruskal–Wallis Test with Dunn’s MCT). (**B**) Expression of *sxp-GAL4/*+ in adult male head visualized with GFP, predominately staining PI neurons and sub-perineural glia. (**C**) Expression of *hts-RD* with *sxp-GAL4* did not rescue *sxp* (****p < 0.0001, n = 20–24, Kruskal–Wallis Test with Dunn’s MCT). (**D**) Compared to *sxp-GAL4/*+ and *UAS-GFP-RNAi/*+ , *UAS-hts-RP-RNAi/*+ was hyperaggressive (**p = 0.0095, n = 12–25, Kruskal–Wallis Test with Dunn’s MCT).

I first tested whether the reduced *hts-RD/RP* in *sxp* males was the cause of their hyperaggressive phenotype. This seemed likely as several *hts* transposons, including a P-Bac insertion 15 Kb 5′ of *CalpA*, were also hyperaggressive (Supplementary Fig. S4). To do this, I attempted to rescue *sxp* with a *hts* genomic construct (*g-hts*) that spans the entire *hts* locus and its regulatory sequences, but not the complete coding sequence of *CalpA*^48^. Indeed, inclusion of the *hts*-genomic construct in the *sxp* mutant, restored normal levels of aggression (Fig. 4A). Therefore, given the nature of the *hts* lesion in *sxp*, I conclude that *hts-RD* and/or *hts-RP* limit the level of isolation-induced aggression, and that reduced *CalpA* is not the cause of its excessive SH aggression.

To try and decipher where *hts-RD* or *hts-RP* regulate aggression I first used *sxp-GAL4*, which is expressed in pars-intercerebralis (PI) neurons and sub-perineural glia (Fig. 4B), and may represent the *hts-RD/RP* expression pattern. However, driving *UAS-hts-RD*^46^ with *sxp-GAL4*, did not normalize *sxp* aggression (Fig. 4C). Therefore, if *hts-RD* is involved, either its expression in these cells was insufficient, or possibly the expression level required for rescue was not optimal. Attempts to rescue by broadening the *sxp-GAL4* expression pattern with either a pan-neuronal or a pan-glia *GAL4* driver failed*,* as all resulted in hyperaggressive *GAL4/*+ control flies, making significant difference between genotypes hard to detect (Supplementary Fig. S4). Similarly, technical limitations also prevented me from testing a role for *hts-RP*, as the available *UAS-hts-RP-RNAi* line was hyperaggressive as a heterozygote (Fig. 4D). In summary, although hyperaggressive control phenotypes precluded me from dissociating the roles of *hts-RD* and *hts-RP* and identifying where they function, my results show that at least one of these *hts* transcripts acts to limit aggression when male flies are isolated.

### Decreasing *CalpA* in *sxp-GAL4* cells increases male–male courtship

**Figure 5:**
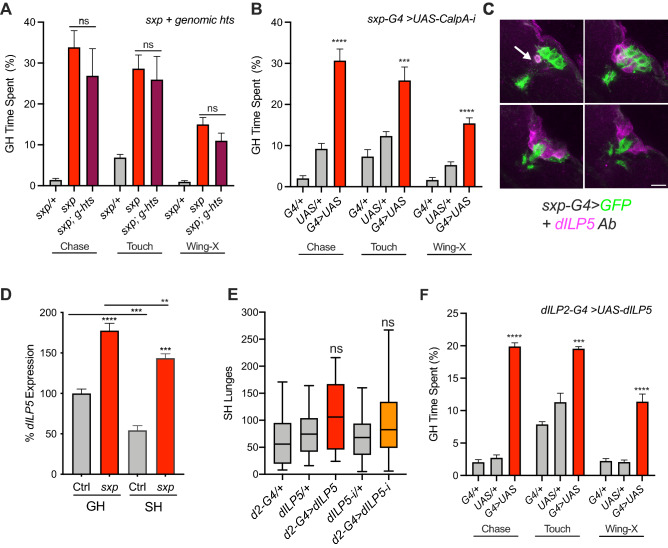
*CalpA* and *dILP5* regulate male–male courtship. (**A**) Expressing *hts* genomic construct (*g-hts*) did not restore normal levels of male–male chasing, touching and single-wing extensions of GH *sxp* in the fly bowl (p > 0.05, n = 4, One-Way ANOVA, with Tukey’s MCT) (**B**) Driving *UAS-CalpA-RNAi* with *sxp-GAL4* increased chase, touch and single-wing extensions in GH flies (****p < 0.0001, ***p < 0.0002, n = 3–6, One-Way ANOVA, with Tukey’s MCT). (**C**) Sub-stacks of a Z-projection showing expression of *sxp-GAL4* neurons visualized with GFP (green) in the PI. Except for one neuron (arrow), GFP expression is intermingled with, but excluded from, DILP5 expressing cells visualized with DILP5 Ab (purple). Scale bar = 10 μm. (**D**) By q-PCR, *dILP5* is overexpressed in GH and SH male *sxp* heads (****p < 0.0001, n = 6). *dILP5* is also reduced by social isolation in both controls and *sxp* (***p < 0.0002, **p < 0.0085, n = 4–6, Two-way ANOVA with Sidak’s MCT). (**E**) Overexpression or reduction of *dILP5* by driving *UAS-dILP5 or UAS-dILP5-RNAi* with *dILP2-GAL4* did not increase SH aggression (p > 0.05, n = 21–24, Kruskal–Wallis Test with Dunn’s MCT). (**F**) Overexpression of *dILP5* with *dILP2-GAL4* did increase male–male chasing, touching and single-wing extensions of GH flies in the fly bowl (****p < 0.0001, **P < 0.0051, n = 4–8, One-way ANOVA with Tukey’s MCT).

Using the *hts* genomic construct (*g-hts*), I next attempted to normalize the excessive male–male courtship of GH *sxp* in the fly bowl. Curiously, unlike aggression, the *hts* genomic construct failed to rescue time spent chasing, touching, and performing single-wing extensions (Fig. 5A). Therefore, I conclude that *hts-RD/RP* does not limit male–male courtship. Given this, I next tested if *CalpA* might regulate male–male courtship by using RNAi to downregulate *CalpA* in *sxp-GAL4* expressing cells*.* Interestingly, driving a *UAS-CalpA-RNAi* with *sxp-GAL4* significantly increased chasing, touching and single-wing extensions in GH males (Fig. 5B, Supplementary Fig. S5). Importantly, as a control for a non-specific RNAi effect, driving an *UAS*-*hts-RNAi* line that reduces all *hts* isoforms with *sxp-GAL4* did not increase single-wing extensions in the fly bowl (Supplementary Fig. S5). These results suggest that *CalpA* functions in *sxp-GAL4* cells to inhibit male–male courtship. Therefore, because the genomic *hts* construct failed to normalize male–male courtship, reduced *CalpA* appears, at least in part, to be responsible for the excessive male–male courtship in GH *sxp* males.

### *dILP5* is overexpressed in *sxp* and increases male–male courtship

The insulin-producing neurons in the PI (PI-IPCs) co-express DILP2, DILP3 and DILP5 and have been shown to regulate aggression, locomotor activity and ethanol resistance (reviewed in^49^). Closer examination of *sxp-GAL4* expression in the PI, revealed that one *sxp-GAL4* neuron was an IPC (Fig. 5C), while the rest (about 14 cells) were intermingled, but exclusive from them. As cells juxtaposed to the PI-IPCs have been implicated in non-autonomously regulating insulin release^50–52^, I next examined levels of brain derived *dILP2, dILP3* and *dILP5* in *sxp* heads by q-PCR. Interestingly, while *dILP2* and *dILP3* expression were unaffected in GH *sxp* (Supplementary Fig. S5), *dILP5* was highly overexpressed in the heads of both GH and SH *sxp* males (Fig. 5D), suggesting an aspect of energy homeostasis is altered in *sxp* males. Curiously, these q-PCR experiments also revealed that social isolation decreases *dILP5* expression, in controls by approximately 50%. This isolation-induced reduction of *dILP5*, is similar to the isolation-induced reduction of *drosulfakinin* (*dsk*)^53^ another neuropeptide also expressed in the PI-IPCs^54^ which regulates aggression^53,55^. However, *dsk* levels in the heads of GH and SH *sxp* males were normal (Supplementary Fig. S5), suggesting *dsk* down regulation by social isolation is normal in *sxp* and not the cause of its excessive SH aggression.

Given the high level of *dILP5* in the heads of *sxp* males, I next tested if overexpressing *dILP5* in the PI-IPCs phenocopied any of its excessive social behaviors. Overexpression of *dILP5* with *dILP2-GAL4* did not increase SH aggression (Fig. 5E). Therefore, it seems likely that *dILP5* overexpression in *sxp* does not increase aggression. This is supported by the lack of aggression in GH *sxp* flies (Fig. 2A), where *dILP5* is also overexpressed (Fig. 5D). However, in the fly bowl, *dILP5* overexpression with *dILP2-GAL4* did increase male–male chasing, touching and single-wing extensions of GH flies (Fig. 5F). Therefore, while *dILP5* overexpression does not appear to promote the excessive aggression of SH *sxp*, it likely contributes to increased male–male courtship.

Finally, as isolation increases aggression as well as reduce *dILP5* expression by 50% in control flies (Fig. 5D), I also tested if knockdown of *dILP5* in PI-IPCs affected SH aggression. However, further reducing *dILP5* by driving *UAS-dilp5-RNAi* with *dILP2-GAL4* in SH flies did not affect aggression (Fig. 5E). Therefore, in control males, it seems unlikely that isolation-induced reduction of *dILP5* increases SH aggression. Furthermore, the isolation-induced reduction of *dILP5* is also unlikely to increase male–male courtship, as this behavior was not strongly induced by isolation (Supplementary Fig. S2).

Taken together, these results suggest reduced *hts-RD/RP* in *sxp* males is the cause of its excessive SH aggression, while increased *dILP5*, and possibly reduced *CalpA*, is the cause of its excessive male–male courtship. Therefore, I conclude that *hts-RD/RP* functions to limit the level of isolation-induced aggression, while *CalpA* inhibits and chronically high *dILP5* increases, male–male courtship.

## Discussion

In this study, I sought to identify genes central to regulating the magnitude of behaviors induced by social isolation. In total, three mutants and candidate genes were identified that affected isolation-induced aggression, locomotor activity and ethanol resistance. However, in none of these mutants was the direction of behavioral regulation consistent across all behaviors (some behaviors were increased, while others decreased), revealing a genetic idiosyncrasy that did not fit a simple model of a single gene acting unidirectionally, in parallel and in different brain regions^34^.

The *sxp* mutant male had decreased activity when isolated and alone, and excessive aggression and ethanol resistance when re-socialized. Regardless of housing regime and in the absence of food and/or more space, *sxp* also had increased male–male courtship. The cause of its unusual behaviors was a P-element insertion that disrupts expression of two neighboring genes, *hts* and *CalpA*, as well as an indirect effect on *dILP5* expression. Specifically, *hts-RD/RP* and *CalpA* were reduced while *dILP5* (on a different chromosome) was increased. Experiments suggest *hts-RD/RP* limits the level of isolation-induced aggression in as yet undefined cells, while *CalpA* functions in *sxp-GAL4* cells and *dILP5* in the PI-IPCs to regulate male–male courtship. It was not specifically tested which gene disruption(s) regulate isolation-induced hyperactivity or ethanol resistance, or if *sxp* females also show abnormal responses to being isolated.

Hts/adducin is a highly expressed protein in the brain^56^, consisting of α/β- or α/γ heterodimers or tetramers that function to bundle actin filaments, cap their growing end, and bind spectrin. In doing so, adducin regulates cytoskeletal dynamics and distribution of plasma membrane proteins. Vertebrates have three *adducin* genes (α-adducin/Add1, β-adducin/Add2, and γ-adducin/Add3) (reviewed in^57^), while *Drosophila* has one (*hts*). Adducin is best known in the nervous system for stabilizing synapses^39,40^, but they also regulate axon guidance^46^ and diameter^58^. Most studies implicate the ‘business end’ of adducin to be its C-terminal MARCKS domain (related to myristoylated alanine-rich C kinase substrate protein)^59^, which is lacking in both *hts-RD* and *hts-RP*. However, *hts-RD* can rescue axon guidance defects in *hts* nulls, suggesting the MARCKS domain is not essential for spectrin binding and possibly other actin related functions^46^. Nothing is known about *hts-RP*, which has a large and unique C-terminal domain. As isolation does not affect *hts-RD/RP* transcription (Supplementary Fig. S3) and because GH *sxp* males are not aggressive, *hts-RD/RP* may also be regulated post-translationally. Indeed, in mice, an enriched environment increases β-adducin phosphorylation^60^.

My attempts to determine in which cells *hts-RD/RP* functions to limit aggression failed, in part because control lines were hyperaggressive. Of note, these hyperaggressive control lines also displayed excessive male–male courtship in the fly bowl, suggesting the presence of food and/or the difference in space between the aggression chamber and fly bowl may have a significant effect on behavior^61,62^. It will be interesting to see if hyperaggressive flies in general show excessive male–male courtship in the absence of food and females. The reason for the hyper-aggression in these control lines is not clear, but as many genes regulate aggression^36^, it could be due to the sum of insertional effects in these randomly inserted *P-GAL4* elements. Alternatively, as not all stocks were backcrossed at the same time, it may also be due to an accumulation of modifiers in parental stocks. Of note 75% of aggression mutants from the original screen were normalized after backcrossing for five generations, suggesting significant effects of time. As *hts-RD/RP* expressing cells were not located, the effect of *hts-RD/RP* reduction on cell morphology and/or neuronal circuitry is yet to be ascertained, though its likely they regulate an aspect of actin remodeling. This could be a developmental effect that is only uncovered when *sxp* is isolated, or an acute failure to alter actin dynamics in response to the change in social environment. Given these findings in the fly, it is noteworthy that in humans, adducin has been associated with bipolar disorder^63^, schizophrenia^64^ and alcohol-use disorders^65^, conditions where emotions can be overpowering.

Calpains are a family of calcium-dependent cysteine proteases with numerous substrates, including adducin^66^, α-spectrin^67^ and the glutamate receptor GluRIIA^68^. In *Drosophila CalpA* regulates immune function^69^ and dendritic pruning^47^, a developmental process also requiring actin remodeling. While the reduction of *CalpA* is relatively small in *sxp* males, it does seem to play a role in inhibiting male–male courtship as the genomic *hts* construct failed to normalize it in *sxp*, while reduction of *CalpA* with *sxp-GAL4* phenocopied it. Interestingly, inhibitors of calpain increase insulin secretion^70^. Therefore, *CalpA* may inhibit *dILP5* expression by decreasing its secretion. Because only one *sxp-GAL4* neuron expressed dILP5 (Fig. 5C), it seems unlikely that a 20% *CalpA* reduction in one cell is increasing *dILP5* expression by ~ 50%. Rather, *CalpA* may function in other *sxp-GAL4*/*dILP5*-negative cells that regulate insulin secretion, such as the juxtaposed fat-sensing PI neurons^50^, the taotie neurons^52^ or even the sub-perineural glia^71^.

Although it was not established which gene disruption(s) in *sxp* results in *dILP5* overexpression, the data suggests *dILP5* does not regulate isolation-induced aggression but, when chronically overexpressed, does promote male–male courtship. As SH *sxp* males are hypoactive and *dILP5* nulls are hyperactive^72^, *dILP5* may also regulate isolation-induced hyperactivity. Since *dILP5* is also reduced during starvation^73^, the reduction of *dILP5* when isolated suggests isolated flies are in some way food deprived. Indeed, although SH flies have a normal appetite^74^, they do weigh less than GH flies (Supplementary Fig. S2). Interestingly, socially isolated ants retain food in their crop (a temporary food storage chamber), preventing its use as an energy source^75^. This mechanism of food retention may therefore explain why SH flies have normal appetite, yet reduced weight and *dILP5* expression. Intriguingly in humans, food and social deprivation activate the same mid-brain region^76^.

In summary, this study identified several candidate genes that regulate sensitivity to being socially isolated. The characterization of *sxp* revealed a molecular and behavioral complexity involving several genes not uncommon in psychiatric disorders^77^ and demonstrated the utility of *Drosophila*^78,79^ in untangling the complex relationships between genetics, the social environment and behavior.

## Materials and methods

All flies were maintained on a 12-h light–dark cycle on standard cornmeal-yeast-agar medium at 25 °C and 60% humidity. All experiments were performed on 7–8-day old males.

### Fly stocks

Fly strains were from the following labs/stock center and I appreciate their generosity. *dILP2-GAL4*, *PDF-GAL4*, *R5710-GAL4*, *Repo-GAL4* and *Repo-GAL80* (Bloomington Stock Center); *dILP3-GAL4*^80^; *UAS-dILP5* (Ernst Hafen, Switzerland), *UAS-hts-RD* (Takashi Suzuki, Japan); *UAS-hts-RNAi* (GD29102), *UAS-hts-RP-RNAi* (KK113568), *UAS-CalpA-RNAi* (GD12275), *UAS-dILP5-RNAi* (GD16039) and fTRG-585^48^ (genomic-hts, VDRC 318186) (VDRC); NP3613 (Kyoto Stock Center) and *C00257* (Harvard). All stocks were backcrossed to *wBerlin* for five generations to remove unlinked modifiers and normalize the genetic background.

### Molecular characterization of *hts* allele

The location of the P-element insertion in *sxp* was determined by inverse PCR. The P-element is located in the first *hts* intron at position 19424308, 515 bp downstream of exon 1. q-PCR was performed on 3–6 biological replicates; each replicate had 16–20, 6-day old male fly heads (three biological replicates/day, three triplicates n = 1). Tissue was homogenized in DNA/RNA shield (Zymo Research) and RNA was extracted using a Direct-zol kit (Zymo Research) and quantified and quality controlled with Nanodrop. 20 ng RNA per sample was used for DNA synthesis and q-PCR using RNA-to-C_T_ 1-Step Kit (Life Technologies). Taqman primers and designed by Applied Biosystems. q-PCR was performed and analyzed by comparative C_T_ methods on a StepOne Plus Machine (Applied Biosystems). Expression levels were normalized and presented in terms of % expression (100% = Relative value = 1).

### Behavior assays

Most behavioral assays were performed on males on 2 independent days using 4–6 independent crosses with the same number of virgins for all crosses (to control for larval density and adult fly size). Single housing involved isolating 1–2 day old flies (non-virgins) in small glass vials for 6–7 days before testing. For group-housed controls 20 males in placed in a regular vial for the same period of time. To control for circadian effects on behavior, all assays were performed within 3 h of lights on. For *GAL4/UAS* experiments, experimental flies were compared with both the *GAL4/*+ and *UAS/*+ controls. Only experimental lines that differed from both controls in the same direction were considered to have a phenotype.

### Aggression

Aggression assays were performed as previously described^81^. Aggression was measured by video recording two flies of the same genotype for 20 min in a 10 mm arena in a 12-cell chamber at 25 °C. The bottom of the chamber was coated with a thin layer of 4% apple juice and agar medium, and walls of the chamber coated with Fluon (BioQuip, Rancho Dominguez, CA). Flies were habituated to the chamber for 5 min before recording. Movies were recorded at 30 frames/s using gVision (http://gvision-hhmi.sourceforge.net) video acquisition software run with Matlab (MathWorks, Natick, MA). Pairs of males were tracked and automated scores of lunging were derived using CADABRA software^82^. The screen controls for aggression were two independent P element lines that had a normal mean/median lunge number (~ 100 lunges/20 min) compared to the population of 1600 strains that were initially screened (Heberlein line *10-195* or *5-116*).

### Locomotor activity

The locomotor/sleep activity was recorded using the Drosophila Activity Monitoring System (Trikinetics, Waltham, MA). Flies were placed in 65 mm × 5 mm glass tubes and acclimated overnight before measurement of sleep/activity. Because all flies are isolated in the trikinetic tubes, the activity/sleep was measured for the first 24 h after acclimation. A sleep bout was defined as 5 min or more of immobility. Total daytime activity/sleep was extracted using S.C.A.M.P software from the Griffith lab. The control for activity was the same strain used for aggression (Line *5-116*) or *w*+ *Berlin,* which were not significantly different.

### Ethanol-induced sedation

Ethanol sedation assays were performed in the “booz-o-mat,” an eight-chambered apparatus that delivers a specified concentration of ethanol vapor^83^. Sedation was assayed manually as described previously^84^ by exposing groups of twenty GH or SH males to an ethanol concentration of 100:50 (ethanol vapor:humidified air). Sedation time is the median time when 50% of flies are sedated. *wBerlin* was used as the control.

### Fly bowl

A detailed account of Fly Bowl assay, data collection and analysis has been previously described^85^. Briefly, flies were gently loaded into the Fly Bowl by aspiration, each bowl had groups of 20 males. I simultaneously recorded 2–4 20 min movies at 25 °C. As 2–4 bowls were loaded before the start of analysis, I took care to rotate genotypes to average out any difference in load time (that ranged from 32 to 482 s as GH flies were quicker to load than SH flies). Analysis was performed using the Janelia Automatic Animal Behavior Annotator (JAABA). I used five behavior classifiers previously described in^85^, using the JAABA interactive machine learning software^86^. Each binary classifier corresponded to a different behavior. These classifiers are not mutually exclusive. A brief description of criteria used to train each behavior classifier is listed: *Chase*—one fly follows another moving fly and maintains a small, somewhat constant distance to the fly it is following. *Touch*—the fly makes contact with the body, wings, or legs of another fly, using its body or legs. *Walk*—the fly moves forward using approximately alternating movements of the mesothoracic legs. *Wing extension*—the fly unilaterally rotates a wing out from its body in the horizontal plane, often for extended periods of time. *Wing-flick*—the fly rapidly and symmetrically moves its wings horizontally out and back to their rest position, often multiple times in a row.

### Immunocytochemistry

Immunocytochemistry was performed using standard techniques on 6–7-day old adult males. Briefly, heads were fixed in 2% PFA o/n at 4 °C before staining o/n in primary antibody. Antibodies used were Anti-GFP (Molecular Probes, A11122, 1:500), Mouse-anti-nc82 (Hybridoma Bank DSHB, 1:50), Anti-FAS-II (DSHB), Anti-Dilp5, 1:800 (courtesy of Prof. P. Leopold), Secondary antibodies were from Molecular Probes and used 1:500.

### Statistics

Statistical analyses were performed using Prism Version 9 (GraphPad). When possible, statistical significance was established by first establishing if there was a normal distribution with a D’Agostino-Pearson normality test. Either a two-tailed t-test or a one-way or two-way analysis of variance (ANOVA), followed by the appropriate post-hoc tests (Unpaired t-test or Tukey’s for normal distribution and a Mann–Whitney or Kruskal–Wallis for non-Gaussian distributions. Alpha = 0.05). Error bars represent min to max in aggression assays and SEM in all other graphs.

## Supplementary Information

Supplementary Figures.

Supplementary Tables.

Supplementary Video S1.

Supplementary Video S2.

## Data Availability

Strains are available upon request. The author affirms that all data necessary for confirming the conclusions of the article are present within the article, figures, and tables.
