# Supplementary material for: A genetic screen for Drosophila social isolation mutants and analysis of sex pistol
Source: Sci Rep. 2021 Aug 30;11:17395. doi: 10.1038/s41598-021-96871-x (PMC8405609; doi:10.1038/s41598-021-96871-x)
Supplement: Supplementary file 1 — Supplementary Figures. [file 41598_2021_96871_MOESM1_ESM.pdf]

A genetic screen for *Drosophila* social isolation mutants and analysis of *sex pistol*

Mark Eddison

Janelia Research Campus

19700 Helix Drive

Ashburn, VA 20147

Email: eddisonm@janelia.hhmi.org

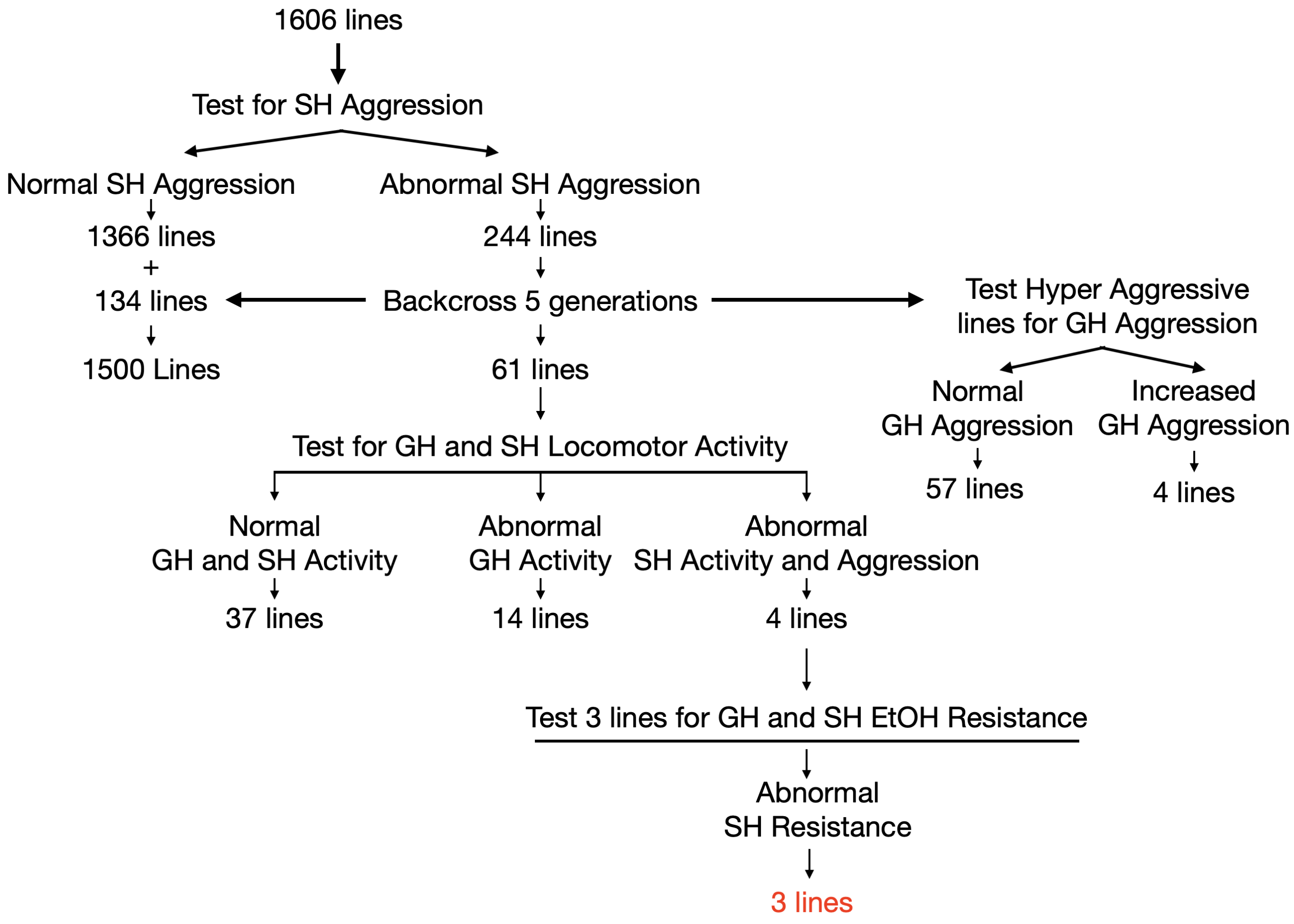

Figure S1) The strategy to identify isolation mutants from a population of 1606 *P-GAL4* insertions

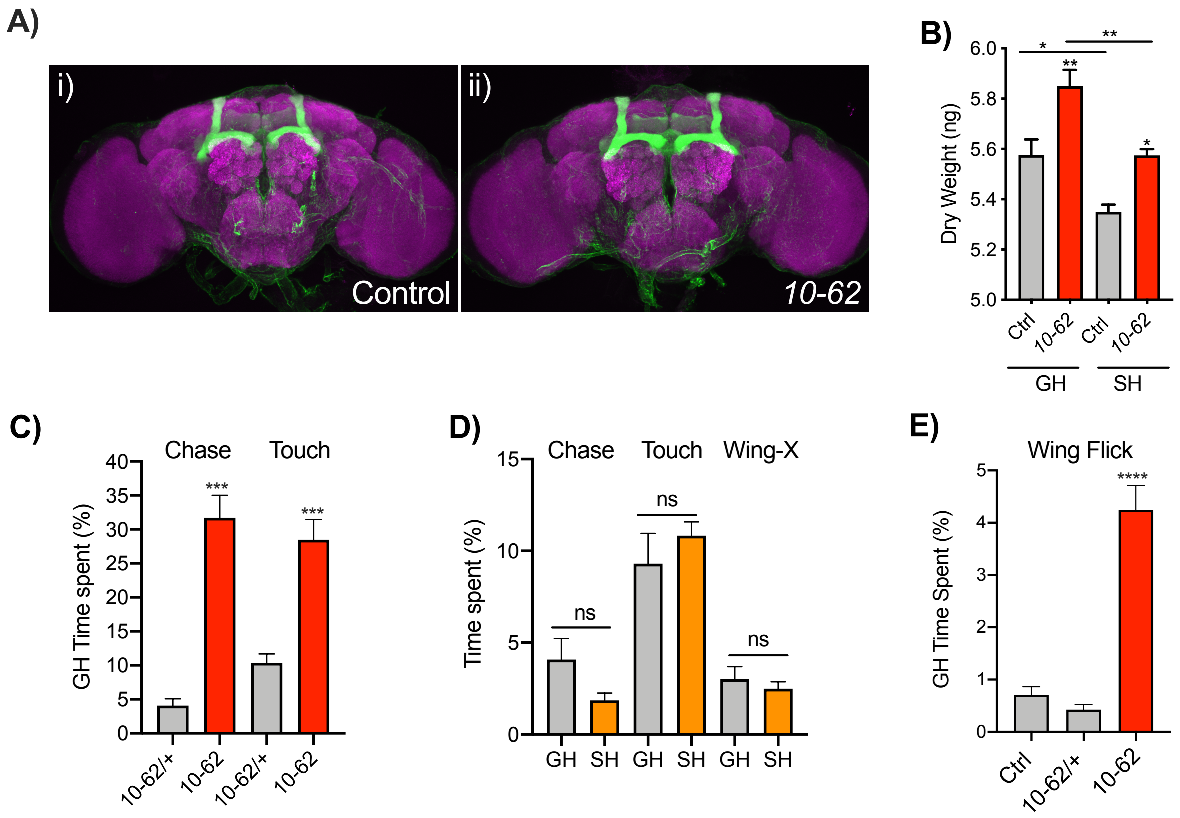

Figure S2) ***10-62* characterization** **(A)** Using Fas2 Antibody (green) to stain the mushroom bodies I detected no major morphological disruptions in the brain of *10-62*. **(B)** *10-62* males weigh significantly more than control males when GH (** $p = 0.0087$) and SH (* $p = 0.0306$). Compared to the GH condition, both control (* $p = 0.0306$) and *10-62* male flies (** $p = 0.0087$) weighed less when SH ($n = 4$, One-Way ANOVA with Tukey's MCT). **(C)** GH *10-62* males also show increased chasing and touching when groups of 20 GH males were placed in the fly bowl ($p < 0.0002$; $n = 8$, Mann-Whitney Test). **(D)** Isolation did not increase chase, touch or single wing extension of screen control flies in the fly bowl ($p > 0.05$; $n = 6$ Unpaired t test). **(E)** GH *10-62* males have increased wing-flicks in the fly bowl (**** $p = 0.0001$; $n = 10$, One-way ANOVA with Tukey's MCT).

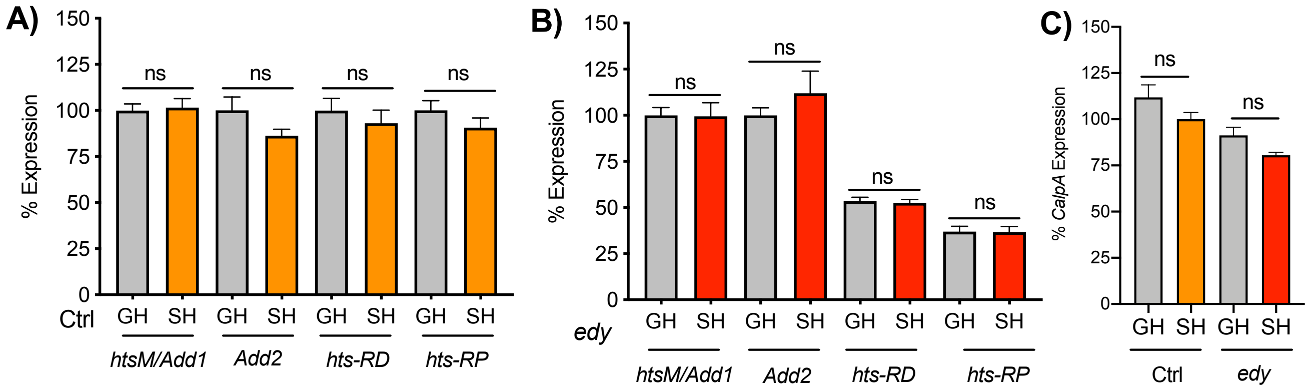

Figure S3) Isolation does not affect *hts* or *CalpA* transcription. Four major *hts* transcripts are unaffected by isolation in both **(A)** control and **(B)** *sxp* flies ($p > 0.05$, $n = 3 – 6$, Unpaired t-test). **(C)** *CalpA* is unaffected by isolation in both control and *sxp* flies ($p > 0.05$, $n = 3$, Unpaired t-test).

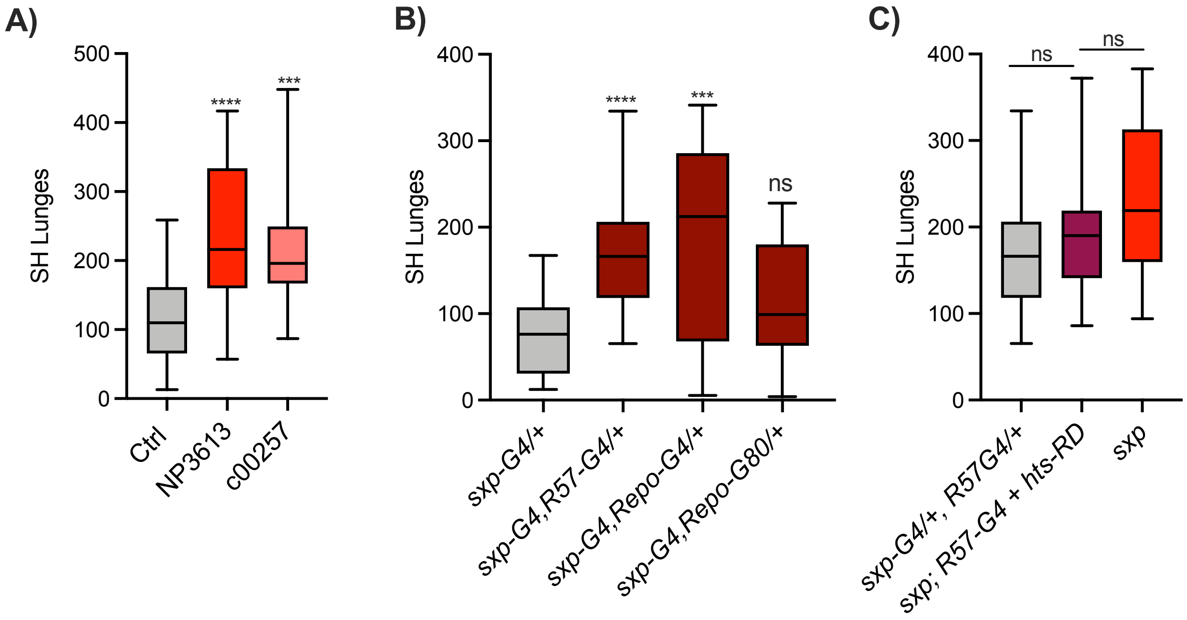

Figure S4) Analysis of *hts* aggression. (A) *hts* P-*GAL4*, *NP3613* that inserts close to *sxp-GAL4*, was hyperaggressive (**** p < 0.0001, n = 22-24) as was *hts* P-Bac *C00257*, that inserts ~15kb 5' of *CalpA* (*** p = 0.0001, n = 24, Kruskal-Wallis Test, with Dunn's MCT). **(B)** The combination of *sxp-G4/+* with *R57C10-GAL4/+* (pan-neuronal), *Repo-GAL4/+* (pan-glia) or *dILP3-GAL4/+* (insulin neurons), but not *Repo-GAL80/+*, increased SH aggression (**** p < 0.0001 n = 20-25, Kruskal-Wallis Test with Dunn's MCT). **(C)** Rescue by pan-neuronal expression of *hts-RD* was inconclusive (p > 0.05, n = 24-25), Kruskal-Wallis Test with Dunn's MCT).

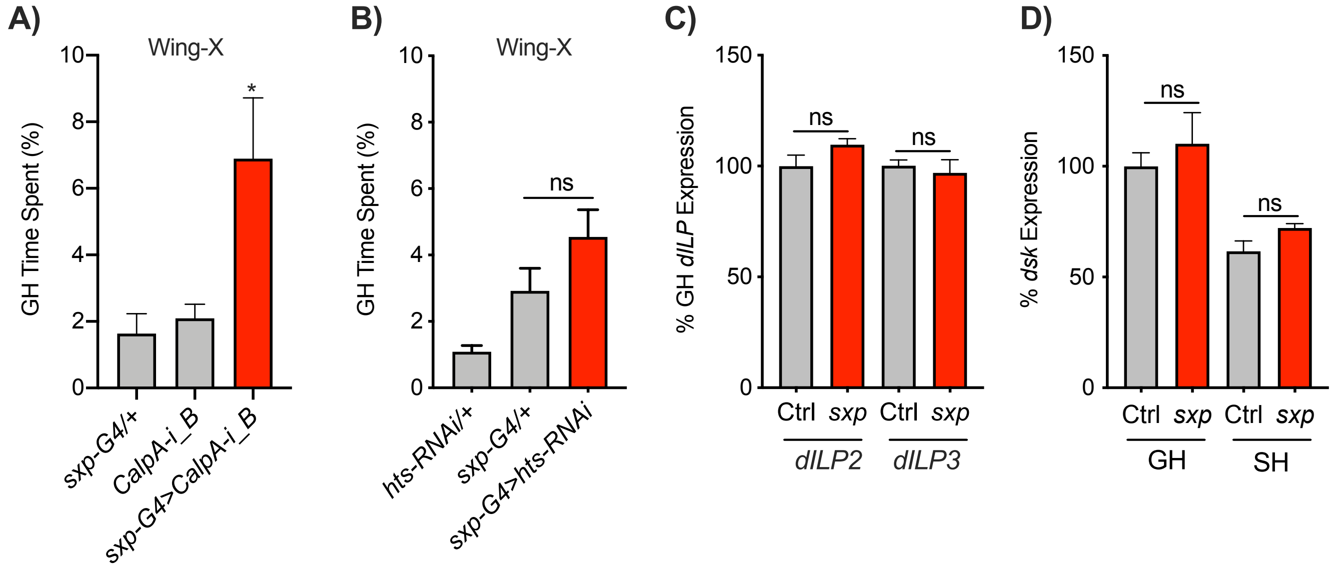

Figure S5) Knockdown of *hts* in *sxp-GAL4* neurons did not increase male-male courtship (A) Driving an independent insertion of *UAS-CalpA-RNAi* with *sxp-G4* increased GH single-wing extensions (* $p < 0.037$, $n = 5$-6, One-way ANOVA with Tukey's MCT). **(B)** Driving a *UAS-hts-RNAi* with *sxp-G4* did not increase single-wing extensions ($p > 0.05$ $n = 8$, One-way ANOVA with Tukey's MCT). **(C)** *dILP2* and *dILP3* expression levels are unaffected in GH *sxp* ($p > 0.05$, $n = 6$, Unpaired t-test). **(D)** *Dsk* expression is unaffected in GH and SH *sxp* ($p = 0.632$, 0.608, $n = 4$, Two-way ANOVA with Sidak's MCT).

Supplementary Tables and Figure Legends

Table S1) Tables of the 46 hyper-aggressive mutants and 15 hypo-aggressive mutants identified from the screen, the P-element insertion site and candidate gene.

Table S2) Tables showing GH and SH daytime activity of SH aggressive mutants.

Table S3) Table showing 4 aggression and activity mutants.

Table S4) Tables showing GH and SH ethanol resistance of 3 aggressive and activity mutants.

Figure S1) The strategy to identify isolation mutants from a population of 1606 *P-GAL4* insertions

Figure S2) *10-62* characterization (A) Using Fas2 Antibody (green) to stain the mushroom bodies I detected no major morphological disruptions in the brain of *10-62*. **(B)** *10-62* males weigh significantly more than control males when GH (** p = 0.0087) and SH (* p = 0.0306). Compared to the GH condition, both control (* p = 0.0306) and *10-62* male flies (** p = 0.0087) weighed less when SH (n = 4, One-Way ANOVA with Tukey's MCT). **(C)** GH *10-62* males also show increased chasing and touching when groups of 20 GH males were placed in the fly bowl ($p < 0.0002$; n = 8, Mann-Whitney Test). **(D)** Isolation did not increase chase, touch or single wing extension of screen control flies in the fly bowl ($p > 0.05$; n = 6 Unpaired t test). **(E)** GH *10-62* males have increased wing-flicks in the fly bowl (**** p = 0.0001; n = 10, One-way ANOVA with Tukey's MCT).

Figure S3) Isolation does not affect *hts* or *CalpA* transcription. Four major *hts* transcripts are unaffected by isolation in both **(A)** control and **(B)** *sxp* flies ($p > 0.05$, n = 3 – 6, Unpaired t-test). **(C)** *CalpA* is unaffected by isolation in both control and *sxp* flies ($p > 0.05$, n = 3, Unpaired t-test).

Figure S4) Analysis of *hts* aggression. **(A)** *hts* P-*GAL4*, *NP3613* that inserts close to *sxp-GAL4*, was hyperaggressive (**** $p < 0.0001$, n = 22-24) as was *hts* P-Bac *C00257*, that inserts ~15kb 5' of *CalpA* (*** p = 0.0001, n = 24, Kruskal-Wallis Test, with Dunn's MCT). **(B)** The combination of *sxp-G4/+* with *R57C10-GAL4/+* (pan-neuronal) or *Repo-GAL4/+* (pan-glia), but not *Repo-GAL80/+*, increased SH aggression (**** $p < 0.0001$ n = 20 -25, Kruskal-Wallis Test with Dunn's MCT). **(C)** Rescue by pan-neuronal expression of *hts-RD* was inconclusive ($p > 0.05$, n = 24-25), Kruskal-Wallis Test with Dunn's MCT).

Figure S5) Knockdown of *hts* in *sxp-GAL4* neurons did not increase male-male courtship (A) Driving an independent insertion of *UAS-CalpA-RNAi* with *sxp-G4* increased GH single-wing extensions (* $p < 0.037$, n = 5-6, One-way ANOVA with Tukey's MCT). **(B)** Driving a *UAS-hts-RNAi* with *sxp-G4* did not increase single-wing extensions ($p > 0.05$ n = 8, One-way ANOVA with Tukey's MCT). **(C)** *dILP2* and *dILP3* expression levels are unaffected in GH *sxp* ($p > 0.05$, n = 6, Unpaired t-test). **(D)** *Dsk* expression is unaffected in GH and SH *sxp* (p = 0.632, 0.608, n = 4, Two-way ANOVA with Sidak's MCT).
